# HIV latency reversal research and the potential effects on the central nervous system: is concern warranted?

**DOI:** 10.7448/IAS.19.1.21008

**Published:** 2016-05-09

**Authors:** Albert M Anderson, Raymond F Schinazi, William R Tyor

**Affiliations:** 1 Division of Infectious Diseases, Department of Medicine, Emory University School of Medicine, Atlanta, GA, USA; 2 Department of Pediatrics, Emory University School of Medicine, Atlanta, GA, USA; 3 Department of Neurology, Emory University School of Medicine, Atlanta, GA, USA; 4 Atlanta Veterans Affairs Medical Center, Atlanta, GA, USA

HIV infection continues to be a significant public health problem with 35 million individuals estimated to be infected worldwide [1]. A curative treatment for HIV infection would be ideal given the problems with access to care, adherence, co-morbidities, drug resistance, aging and costs that persist during combination antiretroviral therapy (cART). However, there has been only one confirmed case of HIV cure worldwide, in which haematopoietic stem cell transplantation was required [2]. Unfortunately, the success of this single transplant has thus far not been replicated in other individuals [3]. A major barrier to HIV cure is that the half-life of resting CD4^+^ T memory lymphocytes, one of the chief sources of HIV latency, has been estimated to be several years [4]. This means that HIV cure will likely not be possible with cART alone. Therefore, cure research strategies have focused on the concept of “kick and kill,” which is based on reactivation of latent HIV from host cells followed by killing of the resultant HIV. Multiple pharmaceuticals based on this strategy are now being studied and have become broadly known as latency reversal agents (LRAs).

As is appropriate for novel research, some have raised theoretical safety concerns about the LRA strategy. Specifically, the central nervous system (CNS) has diminished capacity to regenerate and may be more sensitive than other organs to immune responses during cART [5]. A study presented at the 2015 Conference on Retroviruses and Opportunistic Infections (CROI) suggested CNS injury with combination LRA therapy, albeit in an animal model. This study described three pigtail macaques infected with simian immunodeficiency virus (SIV) on effective cART with undetectable blood SIV levels [6]. While remaining on cART, two of the animals were treated with the protein kinase C activator ingenol-3-hexanoate (Ing-B) in combination with vorinostat, a histone deacetylase (HDAC) inhibitor. One animal quickly developed severe encephalitis requiring euthanasia. The investigators found that this particular animal not only experienced a significant increase in plasma SIV level but also an even more profound increase in CSF SIV level (to 10 times the corresponding level of plasma SIV). The treatment was also associated with significantly higher occipital brain SIV levels in the encephalitic animal, as well as significantly higher levels of CSF neopterin (a marker of immune activation) and neurofilament light chain (a marker of neuronal damage). Despite the fact that the second animal which received active treatment did not exhibit similar changes, the extended observation period before the intervention and the temporal relationship with treatment are suggestive that the outcome in the first animal was treatment induced.

While this study should give investigators pause, it is not clear if the findings are generalizable to humans. Even within macaques, there is evidence that pigtailed macaques (*Macaca nemestrina*) are significantly more susceptible to SIV-associated encephalitis than rhesus macaques (*Macaca mulatta*) [7]. Therefore, the development of encephalitis in this study could be the result of increased susceptibility of the pigtail macaque host. Beyond this, HIV and SIV are different viruses and it is not fully known how mechanisms of latency are similar between the two during cART [8,9].

While still in early stages, studies of LRA in humans have thus far yielded no reports of adverse CNS outcomes. A notable difference is that combination LRA therapy was used in the macaque study, while reports of CNS outcomes in HIV-positive humans during LRA have to our knowledge been limited to monotherapy studies. In a recent CNS substudy of a trial using panobinostat, an HDAC inhibitor, investigators reported no CNS clinical events or significant increase in CSF HIV RNA or inflammatory markers among participants [10]. Another small study of HIV-positive participants (yet to be published as of this writing) showed no significant change in comprehensive neuropsychological testing after treatment with vorinostat [11]. While these early results from LRA interventions in HIV-positive humans are reassuring, the potential for adverse CNS effects during LRA (which may be compounded by combination therapy) should be kept in mind as clinical studies go forward.

An important consideration, particularly with respect to the high incidence of HIV-associated neurocognitive disorders despite cART [12], is the evidence that mononuclear phagocytes (MP) represent a significant reservoir of HIV in the CNS. Viral proteins such as Nef, Tat and Vpr that are expressed early in the HIV replication cycle promote the formation of viral reservoirs in MP by activating transcription and interfering with apoptotic machinery [13]. Viral activation promotes secretion of pro-inflammatory cytokines, which can then lead to chronic inflammation [14]. The dynamic signalling pathways that promote active/infiltrating MP together with the cytokines, chemokines and neurotoxic products they elaborate likely interfere with the homeostatic regulations that maintain normal brain function. Chronic systemic inflammation is tightly linked to morbidity and mortality in cART-treated patients, which suggests that adjunctive anti-inflammatory drugs or immune modulators may improve clinical outcomes [15]. Treatments that combine anti-inflammatory actions with latency reactivation may be the key to safely eradicating HIV, particularly from sensitive sites such as the CNS. A clinical trial sponsored by National Institutes of Health to test the ability of ruxolitinib (a Janus-associated kinase 1/2 inhibitor) to safely modulate the inflammatory effects of HIV will be initiated in 2016 through the AIDS Clinical Trials Group (NCT02475655), with other immune modulating research strategies likely to follow.
